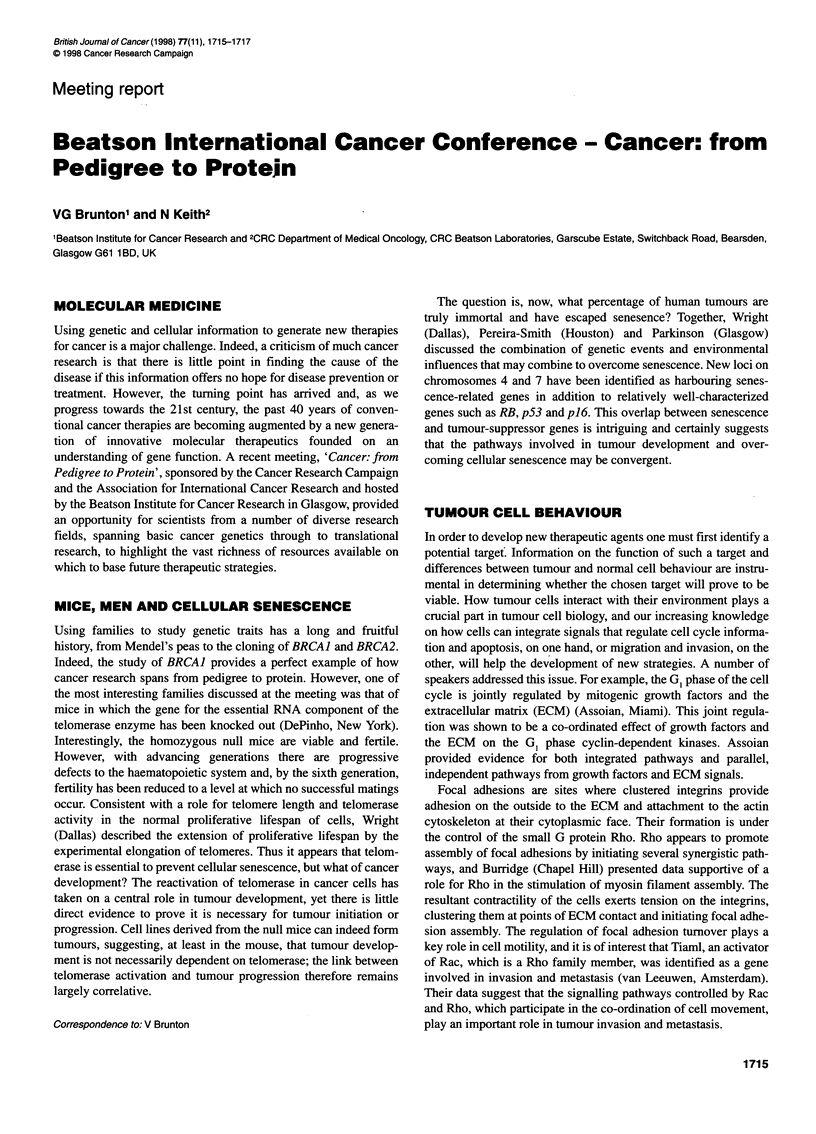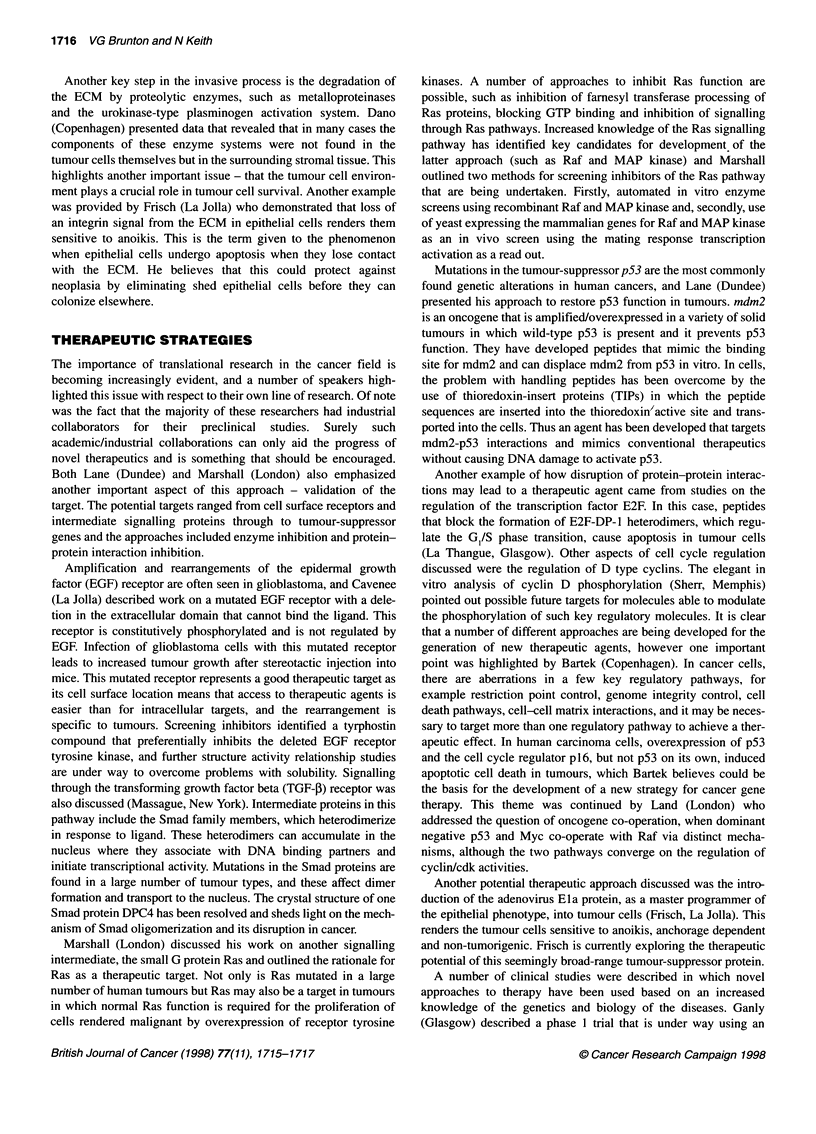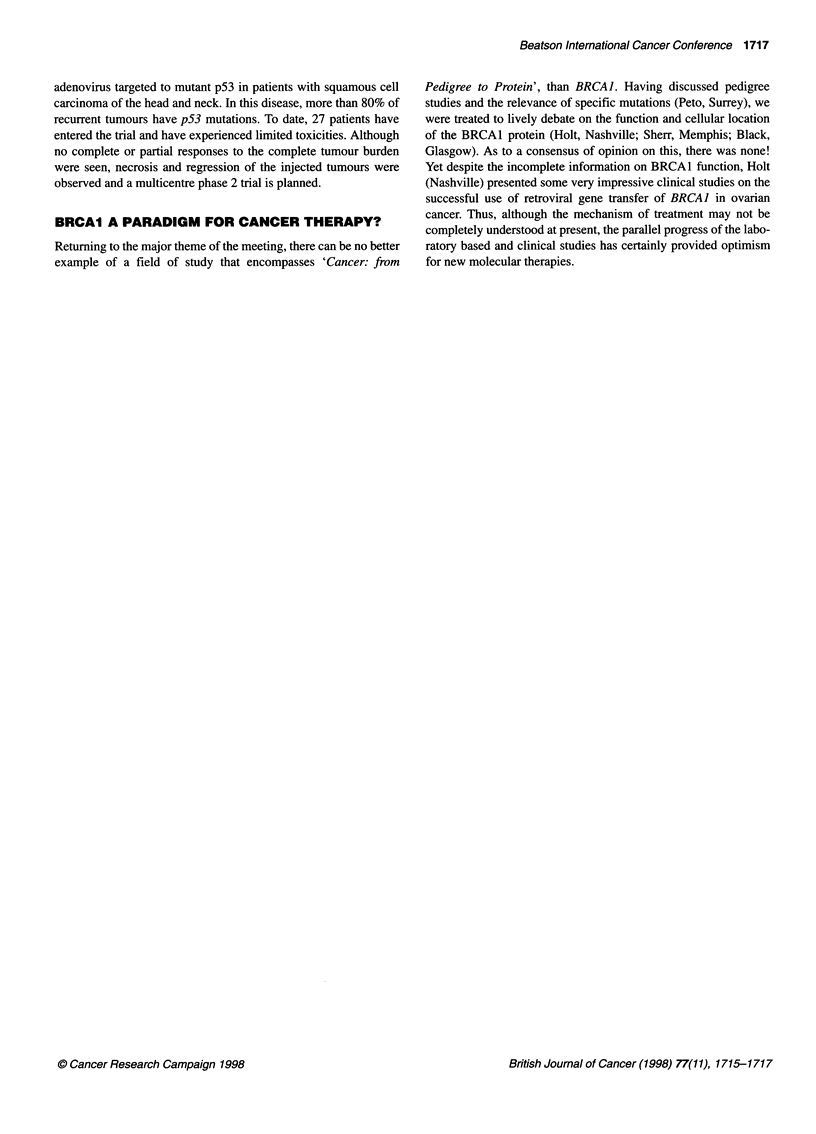# Beatson International Cancer Conference--cancer: from pedigree to protein.

**DOI:** 10.1038/bjc.1998.287

**Published:** 1998-06

**Authors:** V. G. Brunton, N. Keith

**Affiliations:** Beatson Institute for Cancer Research, Glasgow, UK.


British Journal of Cancer (1998) 77(11), 1715–1717
© 1998 Cancer Research Campaign

Meeting report

### Beatson International Cancer Conference – Cancer: from Pedigree to Protein

**VG Brunton[1] and N Keith[2]**

[1]Beatson Institute for Cancer Research and [2]CRC Department of Medical Oncology, CRC Beatson Laboratories, Garscube Estate, Switchback Road, Bearsden, Glasgow G61 1BD, UK

### MOLECULAR MEDICINE

Using genetic and cellular information to generate new therapies for cancer is a major challenge. Indeed, a criticism of much cancer research is that there is little point in finding the cause of the disease if this information offers no hope for disease prevention or treatment. However, the turning point has arrived and, as we progress towards the 21st century, the past 40 years of conventional cancer therapies are becoming augmented by a new generation of innovative molecular therapeutics founded on an understanding of gene function. A recent meeting, '*Cancer: from Pedigree to Protein*', sponsored by the Cancer Research Campaign and the Association for International Cancer Research and hosted by the Beatson Institute for Cancer Research in Glasgow, provided an opportunity for scientists from a number of diverse research fields, spanning basic cancer genetics through to translational research, to highlight the vast richness of resources available on which to base future therapeutic strategies.

### MICE, MEN AND CELLULAR SENESCENCE

Using families to study genetic traits has a long and fruitful history, from Mendel's peas to the cloning of *BRCA1* and *BRCA2*. Indeed, the study of *BRCA1* provides a perfect example of how cancer research spans from pedigree to protein. However, one of the most interesting families discussed at the meeting was that of mice in which the gene for the essential RNA component of the telomerase enzyme has been knocked out (DePinho, New York). Interestingly, the homozygous null mice are viable and fertile. However, with advancing generations there are progressive defects to the haematopoietic system and, by the sixth generation, fertility has been reduced to a level at which no successful matings occur. Consistent with a role for telomere length and telomerase activity in the normal proliferative lifespan of cells, Wright (Dallas) described the extension of proliferative lifespan by the experimental elongation of telomeres. Thus it appears that telomerase is essential to prevent cellular senescence, but what of cancer development? The reactivation of telomerase in cancer cells has taken on a central role in tumour development, yet there is little direct evidence to prove it is necessary for tumour initiation or progression. Cell lines derived from the null mice can indeed form tumours, suggesting, at least in the mouse, that tumour development is not necessarily dependent on telomerase; the link between telomerase activation and tumour progression therefore remains largely correlative.

*Correspondence to:* V Brunton

The question is, now, what percentage of human tumours are truly immortal and have escaped senesence? Together, Wright (Dallas), Pereira-Smith (Houston) and Parkinson (Glasgow) discussed the combination of genetic events and environmental influences that may combine to overcome senescence. New loci on chromosomes 4 and 7 have been identified as harbouring senescence-related genes in addition to relatively well-characterized genes such as *RB*, *p53* and *p16*. This overlap between senescence and tumour-suppressor genes is intriguing and certainly suggests that the pathways involved in tumour development and overcoming cellular senescence may be convergent.

### TUMOUR CELL BEHAVIOUR

In order to develop new therapeutic agents one must first identify a potential target. Information on the function of such a target and differences between tumour and normal cell behaviour are instrumental in determining whether the chosen target will prove to be viable. How tumour cells interact with their environment plays a crucial part in tumour cell biology, and our increasing knowledge on how cells can integrate signals that regulate cell cycle information and apoptosis, on one hand, or migration and invasion, on the other, will help the development of new strategies. A number of speakers addressed this issue. For example, the $G_1$ phase of the cell cycle is jointly regulated by mitogenic growth factors and the extracellular matrix (ECM) (Assoian, Miami). This joint regulation was shown to be a co-ordinated effect of growth factors and the ECM on the $G_1$ phase cyclin-dependent kinases. Assoian provided evidence for both integrated pathways and parallel, independent pathways from growth factors and ECM signals.

Focal adhesions are sites where clustered integrins provide adhesion on the outside to the ECM and attachment to the actin cytoskeleton at their cytoplasmic face. Their formation is under the control of the small G protein Rho. Rho appears to promote assembly of focal adhesions by initiating several synergistic pathways, and Burridge (Chapel Hill) presented data supportive of a role for Rho in the stimulation of myosin filament assembly. The resultant contractility of the cells exerts tension on the integrins, clustering them at points of ECM contact and initiating focal adhesion assembly. The regulation of focal adhesion turnover plays a key role in cell motility, and it is of interest that Tiaml, an activator of Rac, which is a Rho family member, was identified as a gene involved in invasion and metastasis (van Leeuwen, Amsterdam). Their data suggest that the signalling pathways controlled by Rac and Rho, which participate in the co-ordination of cell movement, play an important role in tumour invasion and metastasis.

Another key step in the invasive process is the degradation of the ECM by proteolytic enzymes, such as metalloproteinases and the urokinase-type plasminogen activation system. Dano (Copenhagen) presented data that revealed that in many cases the components of these enzyme systems were not found in the tumour cells themselves but in the surrounding stromal tissue. This highlights another important issue – that the tumour cell environment plays a crucial role in tumour cell survival. Another example was provided by Frisch (La Jolla) who demonstrated that loss of an integrin signal from the ECM in epithelial cells renders them sensitive to anoikis. This is the term given to the phenomenon when epithelial cells undergo apoptosis when they lose contact with the ECM. He believes that this could protect against neoplasia by eliminating shed epithelial cells before they can colonize elsewhere.

### THERAPEUTIC STRATEGIES

The importance of translational research in the cancer field is becoming increasingly evident, and a number of speakers highlighted this issue with respect to their own line of research. Of note was the fact that the majority of these researchers had industrial collaborators for their preclinical studies. Surely such academic/industrial collaborations can only aid the progress of novel therapeutics and is something that should be encouraged. Both Lane (Dundee) and Marshall (London) also emphasized another important aspect of this approach – validation of the target. The potential targets ranged from cell surface receptors and intermediate signalling proteins through to tumour-suppressor genes and the approaches included enzyme inhibition and protein–protein interaction inhibition.

Amplification and rearrangements of the epidermal growth factor (EGF) receptor are often seen in glioblastoma, and Cavenee (La Jolla) described work on a mutated EGF receptor with a deletion in the extracellular domain that cannot bind the ligand. This receptor is constitutively phosphorylated and is not regulated by EGF. Infection of glioblastoma cells with this mutated receptor leads to increased tumour growth after stereotactic injection into mice. This mutated receptor represents a good therapeutic target as its cell surface location means that access to therapeutic agents is easier than for intracellular targets, and the rearrangement is specific to tumours. Screening inhibitors identified a tyrphostin compound that preferentially inhibits the deleted EGF receptor tyrosine kinase, and further structure activity relationship studies are under way to overcome problems with solubility. Signalling through the transforming growth factor beta (TGF-β) receptor was also discussed (Massague, New York). Intermediate proteins in this pathway include the Smad family members, which heterodimerize in response to ligand. These heterodimers can accumulate in the nucleus where they associate with DNA binding partners and initiate transcriptional activity. Mutations in the Smad proteins are found in a large number of tumour types, and these affect dimer formation and transport to the nucleus. The crystal structure of one Smad protein DPC4 has been resolved and sheds light on the mechanism of Smad oligomerization and its disruption in cancer.

Marshall (London) discussed his work on another signalling intermediate, the small G protein Ras and outlined the rationale for Ras as a therapeutic target. Not only is Ras mutated in a large number of human tumours but Ras may also be a target in tumours in which normal Ras function is required for the proliferation of cells rendered malignant by overexpression of receptor tyrosine kinases. A number of approaches to inhibit Ras function are possible, such as inhibition of farnesyl transferase processing of Ras proteins, blocking GTP binding and inhibition of signalling through Ras pathways. Increased knowledge of the Ras signalling pathway has identified key candidates for development of the latter approach (such as Raf and MAP kinase) and Marshall outlined two methods for screening inhibitors of the Ras pathway that are being undertaken. Firstly, automated in vitro enzyme screens using recombinant Raf and MAP kinase and, secondly, use of yeast expressing the mammalian genes for Raf and MAP kinase as an in vivo screen using the mating response transcription activation as a read out.

Mutations in the tumour-suppressor *p53* are the most commonly found genetic alterations in human cancers, and Lane (Dundee) presented his approach to restore p53 function in tumours. *mdm2* is an oncogene that is amplified/overexpressed in a variety of solid tumours in which wild-type p53 is present and it prevents p53 function. They have developed peptides that mimic the binding site for mdm2 and can displace mdm2 from p53 in vitro. In cells, the problem with handling peptides has been overcome by the use of thioredoxin-insert proteins (TIPs) in which the peptide sequences are inserted into the thioredoxin active site and transported into the cells. Thus an agent has been developed that targets mdm2-p53 interactions and mimics conventional therapeutics without causing DNA damage to activate p53.

Another example of how disruption of protein–protein interactions may lead to a therapeutic agent came from studies on the regulation of the transcription factor E2F. In this case, peptides that block the formation of E2F-DP-1 heterodimers, which regulate the $G_1$/S phase transition, cause apoptosis in tumour cells (La Thangue, Glasgow). Other aspects of cell cycle regulation discussed were the regulation of D type cyclins. The elegant in vitro analysis of cyclin D phosphorylation (Sherr, Memphis) pointed out possible future targets for molecules able to modulate the phosphorylation of such key regulatory molecules. It is clear that a number of different approaches are being developed for the generation of new therapeutic agents, however one important point was highlighted by Bartek (Copenhagen). In cancer cells, there are aberrations in a few key regulatory pathways, for example restriction point control, genome integrity control, cell death pathways, cell–cell matrix interactions, and it may be necessary to target more than one regulatory pathway to achieve a therapeutic effect. In human carcinoma cells, overexpression of p53 and the cell cycle regulator p16, but not p53 on its own, induced apoptotic cell death in tumours, which Bartek believes could be the basis for the development of a new strategy for cancer gene therapy. This theme was continued by Land (London) who addressed the question of oncogene co-operation, when dominant negative p53 and Myc co-operate with Raf via distinct mechanisms, although the two pathways converge on the regulation of cyclin/cdk activities.

Another potential therapeutic approach discussed was the introduction of the adenovirus E1a protein, as a master programmer of the epithelial phenotype, into tumour cells (Frisch, La Jolla). This renders the tumour cells sensitive to anoikis, anchorage dependent and non-tumorigenic. Frisch is currently exploring the therapeutic potential of this seemingly broad-range tumour-suppressor protein.

A number of clinical studies were described in which novel approaches to therapy have been used based on an increased knowledge of the genetics and biology of the diseases. Ganly (Glasgow) described a phase 1 trial that is under way using an

© Cancer Research Campaign 1998

adenovirus targeted to mutant p53 in patients with squamous cell carcinoma of the head and neck. In this disease, more than 80% of recurrent tumours have *p53* mutations. To date, 27 patients have entered the trial and have experienced limited toxicities. Although no complete or partial responses to the complete tumour burden were seen, necrosis and regression of the injected tumours were observed and a multicentre phase 2 trial is planned.

### BRCA1 A PARADIGM FOR CANCER THERAPY?

Returning to the major theme of the meeting, there can be no better example of a field of study that encompasses '*Cancer: from Pedigree to Protein*', than *BRCA1*. Having discussed pedigree studies and the relevance of specific mutations (Peto, Surrey), we were treated to lively debate on the function and cellular location of the BRCA1 protein (Holt, Nashville; Sherr, Memphis; Black, Glasgow). As to a consensus of opinion on this, there was none! Yet despite the incomplete information on BRCA1 function, Holt (Nashville) presented some very impressive clinical studies on the successful use of retroviral gene transfer of *BRCA1* in ovarian cancer. Thus, although the mechanism of treatment may not be completely understood at present, the parallel progress of the laboratory based and clinical studies has certainly provided optimism for new molecular therapies.

© Cancer Research Campaign 1998